# Supplementary figures and images for: Decoupling social status and status certainty effects on health in macaques: a network approach
Source: PeerJ. 2016 Sep 13;4:e2394. doi: 10.7717/peerj.2394 (PMC5028790; doi:10.7717/peerj.2394)

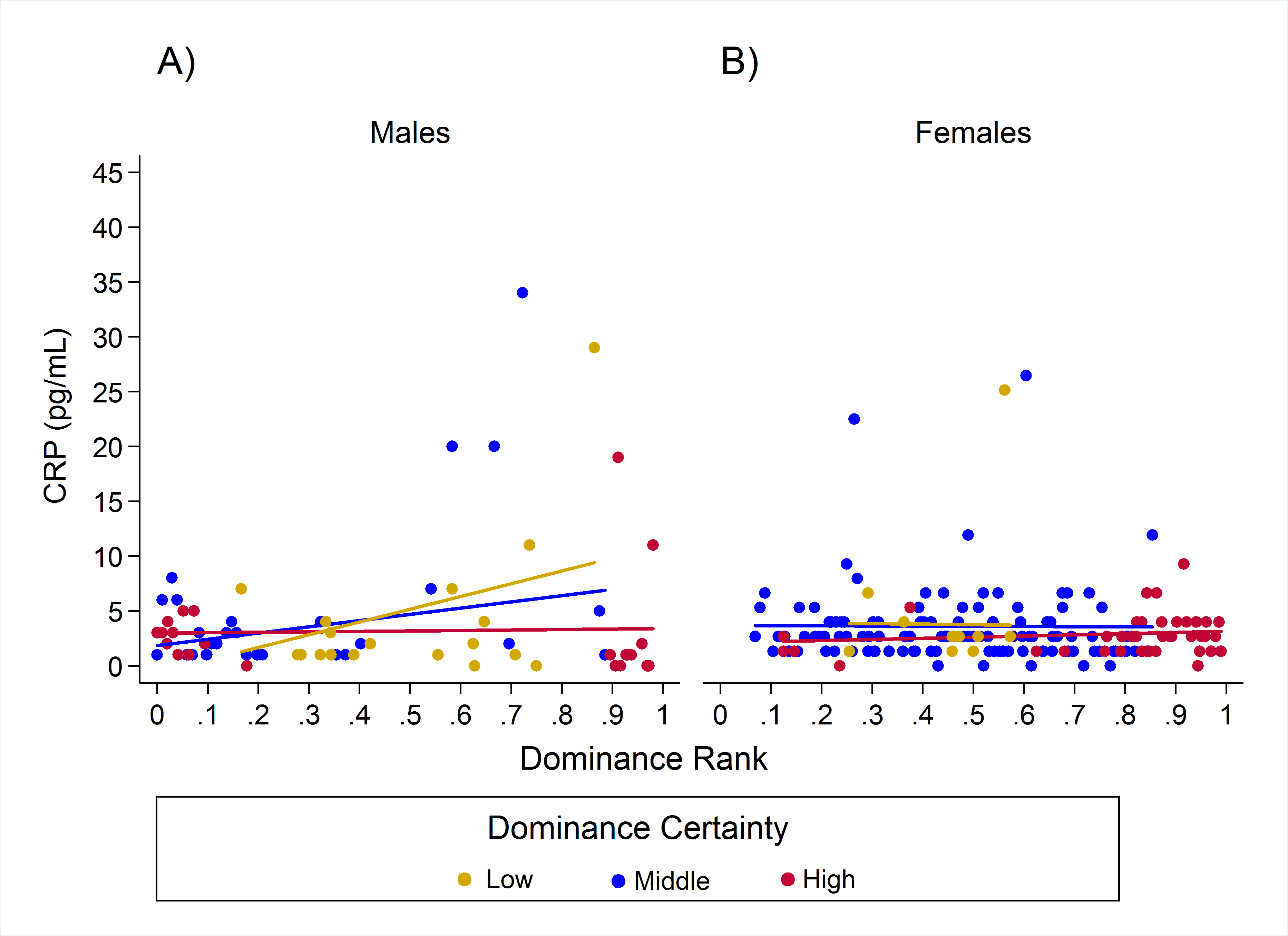

Supplement: Supplemental Information 2 — Raw values of CRP. A and B depict effects for CRP for males and females, respectively. Separate lines represent different levels of dominance certainty. [file peerj-04-2394-s002.png]

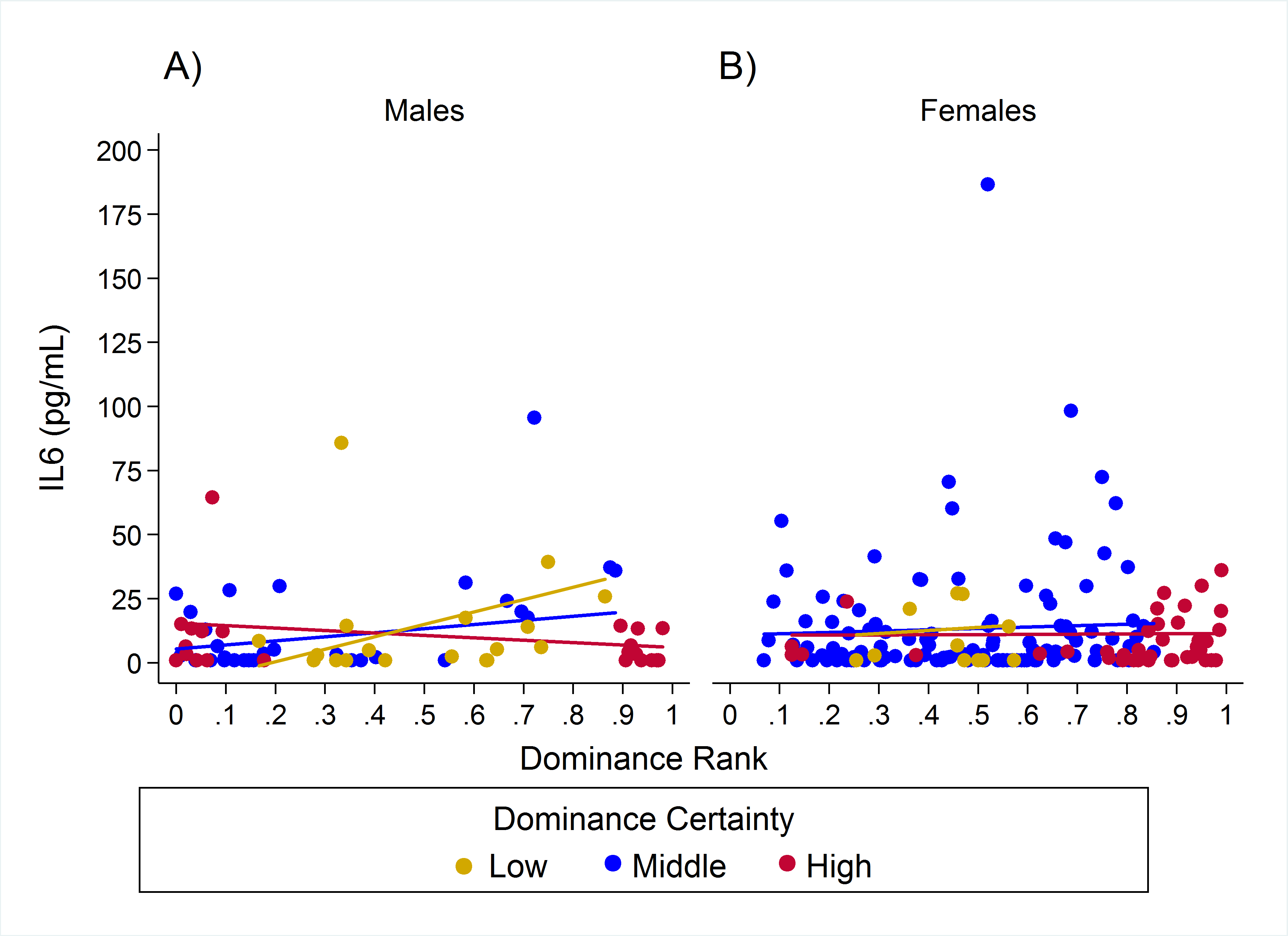

Supplement: Supplemental Information 3 — Raw values of IL-6. A and B depict effects for CRP for males and females, respectively. Separate lines represent different levels of dominance certainty. [file peerj-04-2394-s003.png]

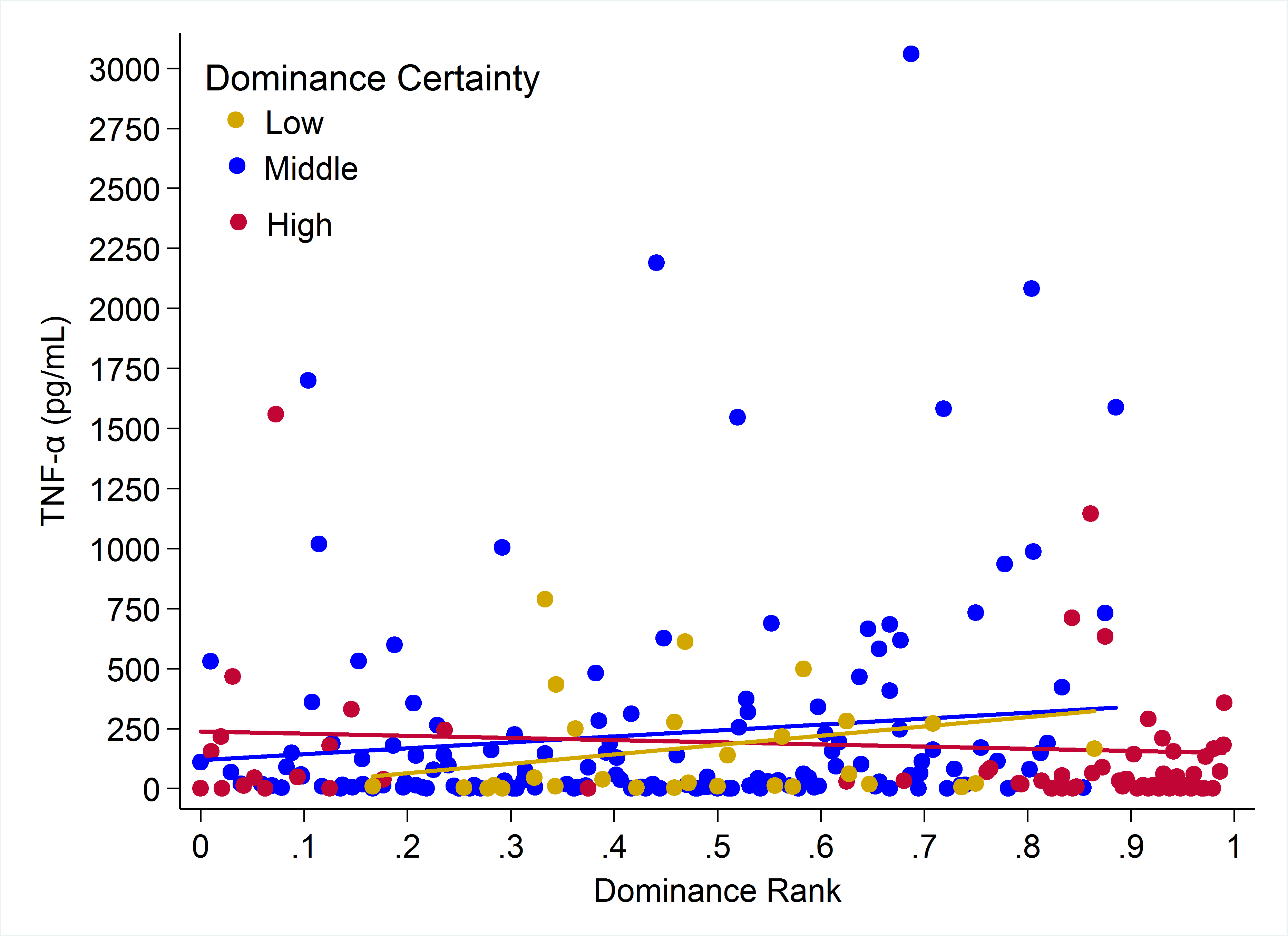

Supplement: Supplemental Information 4 — Raw values of TNF-α plotted against dominance rank. Separate lines represent different levels of dominance certainty. [file peerj-04-2394-s004.png]
